# Deep Brain Stimulation for Treatment-Resistant Depression: Towards a More Personalized Treatment Approach

**DOI:** 10.3390/jcm9092729

**Published:** 2020-08-24

**Authors:** Milaine Roet, Jackson Boonstra, Erdi Sahin, Anne E.P. Mulders, Albert F.G. Leentjens, Ali Jahanshahi

**Affiliations:** 1 Department of Neurosurgery, Maastricht University Medical Center, P. Debyelaan 25, 6202 AZ Maastricht, The Netherlands; m.roet@maastrichtuniversity.nl (M.R.); j.boonstra@maastrichtuniversity.nl (J.B.); a.mulders@maastrichtuniversity.nl (A.E.P.M.); 2 European Graduate School of Neuroscience (EURON); 6229 ER Maastricht, The Netherlands; a.leentjens@maastrichtuniversity.nl; 3 Department of Neurology, Istanbul Faculty of Medicine, Istanbul University Capa, Istanbul 34093, Turkey; erdisahin@gmail.com; 4 Department of Psychiatry and Psychology, Maastricht University Medical Center, P. Debyelaan 25, 6202 AZ Maastricht, The Netherlands

**Keywords:** major depressive disorder, treatment resistant depression, deep brain stimulation, neuropsychological subtypes, personalized treatment approach

## Abstract

Major depressive disorder (MDD) affects approximately 4.4% of the world’s population. One third of MDD patients do not respond to routine psychotherapeutic and pharmacotherapeutic treatment and are said to suffer from treatment-resistant depression (TRD). Deep brain stimulation (DBS) is increasingly being investigated as a treatment modality for TRD. Although early case studies showed promising results of DBS, open-label trials and placebo-controlled studies have reported inconsistent outcomes. This has raised discussion about the correct interpretation of trial results as well as the criteria for patient selection, the choice of stimulation target, and the optimal stimulation parameters. In this narrative review, we summarize recent studies of the effectiveness of DBS in TRD and address the relation between the targeted brain structures and clinical outcomes. Elaborating upon that, we hypothesize that the effectiveness of DBS in TRD can be increased by a more personalized and symptom-based approach. This may be achieved by using resting-state connectivity mapping for neurophysiological subtyping of TRD, by using individualized tractography to help decisions about stimulation target and electrode placement, and by using a more detailed registration of symptomatic improvements during DBS, for instance by using ‘experience sampling’ methods.

## 1. Introduction

Major depressive disorder (MDD) is a common mood disorder that affects one’s feelings, thoughts, and behavior. According to the DSM-5, for a diagnosis of MDD, five of the following symptoms need to be present for at least two weeks: depressed mood, reduced interest or pleasure, weight loss or reduced appetite, insomnia or hypersomnia, psychomotor agitation or retardation, fatigue or loss of energy, worthlessness or excessive guilt, impaired concentration or indecisiveness, and recurrent thoughts of death or suicidal ideation or attempts. Either ‘depressed mood’ or ‘loss of interest or pleasure’ is essential for a diagnosis [1]. The total number of people suffering from MDD worldwide was estimated to be 322 million in 2015 and its prevalence increased by 18.4% between 2005 and 2015 [2]. Therefore, effective treatment of MDD merits intense consideration. 

Whereas psychotherapy and antidepressant medication are effective in the majority of patients, approximately one third of patients do not respond to these therapies. In the Sequenced Treatment Alternatives to Relieve Depression (STAR*D) trial, the cumulative remission rate of MDD patients after four successive treatments was 67% [3]. In line with this, a meta-analysis of 92 studies of the effectiveness of psychotherapy showed that 62% of patients no longer met the criteria of depression after treatment [4]. Failure to respond to a treatment algorithm of several steps is commonly referred to as treatment resistance, although there is still discussion about the exact definition of treatment refractoriness [5]. Treatment-resistant depression (TRD) is associated with more (comorbid) mental health disorders, a higher number of hospitalizations, and more suicide attempts, leading to higher treatment costs compared to non-TRD [6]. In addition, patients with TRD show a higher demand of healthcare resources and costs of health care compared to non-TRD patients [7]. Various alternative treatment options for TRD are currently being investigated, including vagal nerve stimulation [8], repetitive transcranial magnetic stimulation (rTMS) [9], and deep brain stimulation (DBS) [10]. 

The aim of this narrative review is to provide an overview of recent studies of the effectiveness of DBS in TRD with a special focus on the relationship between the targeted brain structures and clinical outcomes. Based on these findings, we discuss the importance of distinguishing between different clinical phenotypes of depression that would allow for more personalized symptom-based treatment approaches, which may be a key factor in improving treatment outcomes.

## 2. Recent Insights on the Pathophysiology of Depression

**Figure 1:**
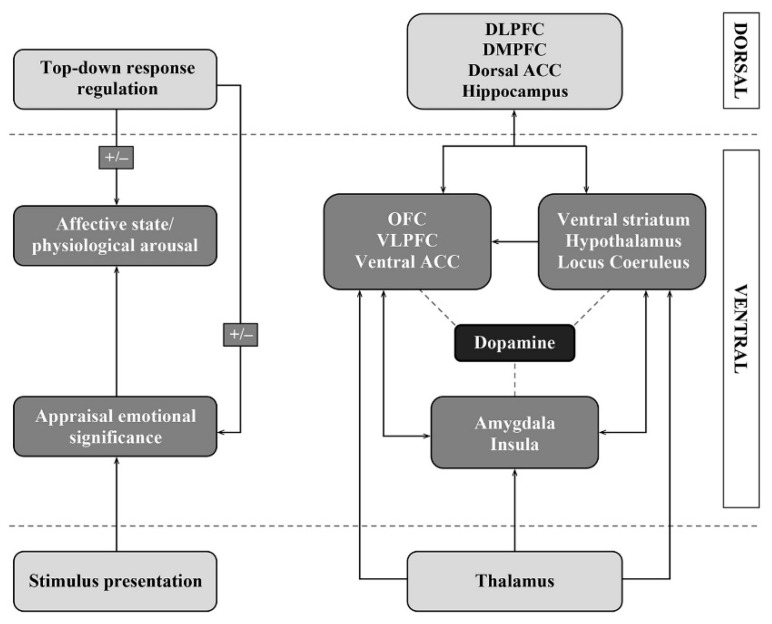
Schematic representation of emotional processing and its neurobiological base. Figure from Moonen et al. (2017) [16] with permission.

It is hypothesized that in depression, there is an imbalance in the limbic cortico-striatal-thalamo-cortical (CSTC) mood circuits [11,12], yet many aspects of circuitopathy in MDD remain largely unknown. Based on different models [11,12], three main components of the CSTC mood circuits have been proposed (Figure 1). First, the ventral component is essential for recognizing emotions and initiating an adequate emotional and behavioral response. In this circuit, the amygdala, ventral striatum, ventral part of the anterior cingulate cortex, orbitofrontal cortex, ventrolateral prefrontal cortex, and downstream structures such as the hypothalamus and locus coeruleus are involved. Second, the dorsal component that regulates the emotional responses and requires cognitive processing. Here, the dorsolateral and dorsomedial prefrontal cortex, the dorsal part of the anterior cingulate cortex, and the hippocampus are involved. Third, a modulating region is present, although no consensus has been made about its precise anatomical organization and function. Some have suggested that this component consists of the thalamus and the rostral part of the anterior cingulate cortex [11,12,13]. As implied by Mayberg et al., the model of depression indicates that depression is associated with a decreased activity in dorsal limbic and neocortical regions and a relative increase in ventral paralimbic regions. Treatment of depression therefore requires the inhibition of the overactive ventral regions, resulting in the disinhibition of the underactive dorsal regions. To mediate this process, proper functioning of the rostral cingulate cortex is required [12]. These mood circuits overlap with the circuitry involved in compulsive traits; DBS of the ventral capsule/ventral striatum (VC/VS) in treatment resistant obsessive-compulsive disorder (OCD) patients has led to improvements in mood which prompted studying the application of DBS in TRD patients [14,15].

### Expanding the Cortico-Striatal-Thalamo-Cortical Mood Circuits

**Figure 2:**
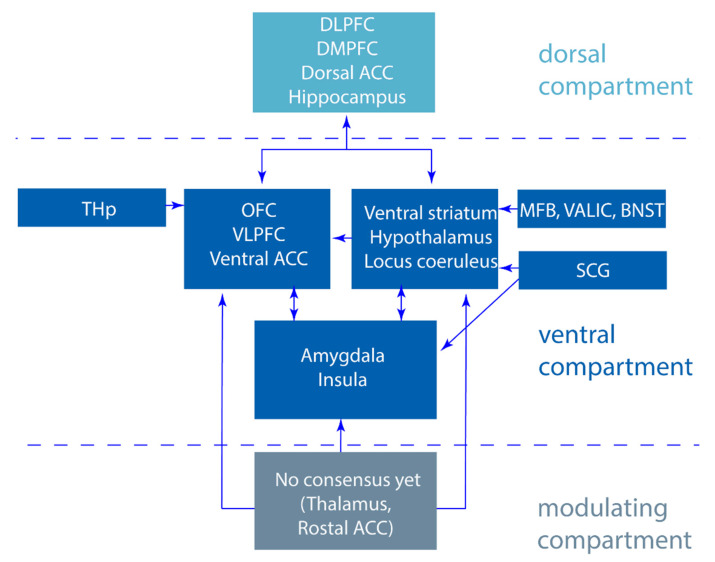
Cortico-striatal-thalamic-cortico mood circuits divided in a dorsal, ventral, and modulating compartment based on Alexander et al. [11], Mayberg et al. [12], and Moonen et al. [16] expanded with regions researched with deep brain stimulation (DBS) for treatment-resistant depression (TRD). DLPFC; dorsolateral prefrontal cortex, DMPFC; dorsomedial prefrontal cortex, ACC; anterior cingulate cortex, THp; thalamic peduncles, OFC; orbitofrontal cortex, VLPFC; ventrolateral prefrontal cortex, MFB; medial forebrain bundle, vALIC, ventral part of the anterior limb of the internal capsule, BNST; bed nucleus of the stria terminalis, SCG; subgenual cingulate gyrus, HPA axis; hypothalamic pituitary adrenal axis.

One region that is not included in the CSTC mood circuits and yet has been a region of interest for DBS targeting in depression for over a decade is the subgenual cingulate gyrus/cortex (SCG/SCC) [10]. This region has shown hyperactivity in untreated depressed patients [17], is part of the ventral component, and has projections to the amygdala, hippocampus, superior and medial temporal gyri, ventral striatum, mid- and posterior cingulate cortex, thalamus, hypothalamus, periaqueductal gray, and lateral habenula [18,19]. Furthermore, in recent years, it has become known that several other brain areas all belonging to the ventral component play a role in the pathophysiology of depression. Among these are the thalamic peduncles (THp) that interconnects with the prefrontal cortex including the orbitofrontal cortex (OFC) [20], the medial forebrain bundle (MFB) that projects to the frontal cortex, the NAcc and ventral striatum [21], and the ventral part of the anterior limb of the internal capsule (vALIC) which forms a homeostatic system with the MFB and the bed nucleus of the stria terminalis (BNST) [22] (Figure 2).

## 3. Deep Brain Stimulation for Treatment-Resistant Depression

DBS is an invasive neuromodulation technique that is effective in managing clinical symptoms of neurological and psychiatric disorders, such as Parkinson’s disease (PD) [23,24] and OCD [25]. At stimulation settings commonly used in clinical practice, DBS decreases the spontaneous firing of neuronal populations and activates axonal projections near the electrode [26]. This modulates pathological activity and replaces it with regular patterns of discharge with intervals of burst activity [27,28]. More recent theories suggest that DBS destabilizes abnormal synchronous oscillatory activity within the basal ganglia circuitry improving hyperkinetic symptomology [24]. However, the exact mechanism(s) by which DBS normalizes electrical activity in the basal ganglia and exerts beneficial effects on PD symptoms remain unknown. In DBS for TRD, target selection has mostly been based on either neuroimaging studies or clinical observations of mood improvement following DBS in OCD [10,15,29]. For these reasons, the underlying mechanisms of action are poorly studied. DBS studies for TRD (Table 1) and the outcomes for selected brain targets (Table 2) are described below.

### 3.1. Subgenual Cingulate Gyrus/Cortex

The first clinical trial of DBS of the SCG for TRD was performed in 2005 and included six patients with MDD [10]. The severity of depression was measured using the Hamilton Depression Rating Scale (HDRS) and the Montgomory Asberg Depression Rating Scale (MADRS). The HDRS has been the gold standard for the assessment of depression for years [51]. A clinical response is commonly defined as a decrease in the HDRS score of more than 50% compared to baseline, and clinical remission is defined as a decrease in the HDRS score to eight or less. After one month, two out of six patients met the criteria for response. At the end of the sixth month, a response was seen in four out of six patients, with three of the patients reaching remission or near remission. Preliminary observations with positron emission tomography (PET) showed a metabolic hyperactive SCG (Brodmann area 25, Cg 25) during depressive states. It was speculated that DBS would reduce this hyperactivity [17] (Table 2). The improvement in depression scores after DBS was thought to be due to effectively disrupting focal pathological activity in limbic-cortical circuits. After 3 months of stimulation of the subgenual cingulate region (CG25) in patients suffering from TRD, local cerebral blood flow (CBF) was decreased in CG25 and the adjacent orbitofrontal cortex (Brodmann area 11). Moreover, after three and six months of stimulation, CBF was decreased in the hypothalamus, anterior insula, and medial frontal cortex of long-term responders, while CBF increased in the dorsolateral prefrontal cortex (dlPFC), dorsal anterior, posterior cingulate, and premotor and parietal regions (Table 2) [10]. In the different open-label trials, response rates varied from 20 to 57% after 1 month, 33.3 to 87.5% after 6 months, and 29 to 62.5% after 12 months (Table 1) [10,33,49,52,53,54,55,56,57,58,59,60,61]. In a long term follow-up, Kennedy et al. (2011) reported response rates at 1, 2, and 3 years after DBS implantation in the SCC of TRD patients of 62.5%, 46,2%, and 75%, respectively [52] (Table 1). In a case series of DBS of the SCG in five TRD patients, a decrease in the score of the depression rating scale was only found in one of the five TRD patients. This patient turned out to be stimulated in the posterior gyrus rectus (PGR) based on single subject tractography results rather than the initially targeted CG25 [62]. A recent exploratory meta-analysis of four observational studies investigating DBS for TRD (Holtzheimer et al. 2012, Lozano et al. 2012, Puigdemont et al. 2012, and Kennedy et al. 2011) reported relatively large response and remission rates following DBS treatment: the twelve-month response and remission rates were 39.9% (95% CI = 28.4% to 52.8%) and 26.3% (95% CI = 13% to 45.9%). The included studies reported a significant decrease in depression scores between 3 and 6 months (Hedges’ g = −0.27, *p* = 0.003), while no additional decrease was found between 6 and 12 months, suggesting that maximal antidepressant effects occur mostly within the first 6 months of treatment [63]. However, adverse events can occur, including worsening of depression, suicidal ideation, and seizures (Table 1). A study consisting of a double-blind active vs. sham stimulation phase of four weeks, followed by an open-label stimulation for up to 24 months, reported no significant differences between the active and sham stimulation of the SCG and no reduction in HDRS scores in the first four weeks. In the open-label phase, response rates were 37.5%, 43% and 23% after 6, 12 and 28 months, respectively. Remission rates were 12.5% and 14.2% at 6 and 12 months, respectively, and 33.3% at 24 and 28 months [64]. 

A randomized controlled trial (RCT) investigating DBS of the subcallosal cingulate, known as the BROADEN trial, was aborted prematurely. The study lasted six months, during which all patients should have received SCC implantation surgery. After six months, blinding would have been uncovered and both groups would have been offered open-label DBS for another six months. At the end of the first six months, responses of the treatment group and control group were predicted to be 40% and 18.5%, respectively. In this trial, the response rate was defined as more than or equal to a 40% decrease in MADRS scores from baseline. However, after six months, only 20% of patients (*n* = 12) in the treatment group showed a response versus 17% of patients (*n* = 5) in the control group. At that time, a futility analysis predicted the probability of a successful study outcome to be 17% or less leading to the funding for DBS electrodes for this study to be discontinued. The actual study was never published, but results were published and mentioned in Morishita et al. (2014) [65,66]. It has been postulated that the patients enrolled in the BROADEN trial had extreme and chronic depression with a mean duration of the current depressive episode of 12 years, nearly twice that of previous open-label studies. Therefore, these patients could have required a longer treatment period before significant results emerge. Long-term outcomes of SCG DBS in TRD patients for up to 8 years show that most patients have a sustained antidepressant response [60]. However, these results need to be interpreted carefully as the patient group consisted of both MDD and bipolar type-II disorder patients. Further comparison between high- and low frequency DBS in the SCG in TRD showed no significant difference in effectiveness between the two groups and a 44.44% response rate at 13 months of stimulation [67].

### 3.2. Nucleus Accumbens

Another brain region involved in MDD is the nucleus accumbens (NAcc), part of the mesolimbic dopaminergic circuit involved in different cognitive functions such as motivation and reward [33] (Table 2). DBS of the NAcc exerts immediate and long-term positive clinical effects in TRD and has been shown to significantly improve depression scores within one week [33]. Visualized with PET–computed tomography (PET-CT or PET/CT), NAcc-DBS increased metabolic activity in the ventral striatum, dlPFC, dorsomedial PFC (dmPFC), cingulate cortex, and the amygdala. Furthermore, metabolic activity in the vmPFC, ventrolateral prefrontal cortex (vlPFC), dorsal caudate nucleus, and part of the thalamus were decreased. Targeting the NAcc was essential for the effect of DBS on anhedonia (i.e., the inability to feel pleasure) in patients suffering from TRD. However, when Schlaepfer et al. (2008) looked at single items of depression rating scales, capturing aspects of anhedonia such as ‘work and activities’, ‘apparent sadness’, and the ‘inability to feel’, no significant improvements were found following NAcc-DBS. A follow-up study showed a 50% response rate in 10 patients suffering from TRD undergoing NAcc-DBS after 10 months [53]. In a more recent study reporting the long term effects of NAcc-DBS, 45% of TRD patients (*n* = 11) were classified as responders with a 50% reduction in HDRS scores after 12 months of stimulation, which remained until the last follow-up of 4 years [54] (Table 1). Several side effects were reported, such as seizure, agitation, and a transient increase in anxiety. In addition, one attempted suicide and one completed suicide were reported, for which the relation with the DBS treatment is uncertain.

### 3.3. Ventral Capsule/Ventral Striatum

The ventral capsule/ventral striatum (VC/VS) is thought to be hyperactive in MDD [36] (Table 2). Capsulotomy (i.e., lesioning) of the VC/VS improved not only OCD symptoms but also depressive symptoms, inspiring stimulation of the VC/VS for TRD [15]. In an open-label trial that stimulated the VC/VS in 15 TRD patients, responder rates at three months, six months, and 12 months were 53.3%, 46.7%, and 53.3%, respectively, using the MADRS as an outcome measure, and were 46.7%, 40%, and 53.3%, respectively, using the HDRS as an outcome measure [55]. Adverse events ranged from pain or discomfort at the incision site, to hypomania, mixed bipolar state, and increased depression due to battery depletion.

The first RCT of DBS of the VC/VS for TRD was performed by Dougherty et al. (2015) who stimulated 30 patients for 16 weeks. There were no significant differences in response rates between the intervention and sham group in the double-blind phase [68,69]. Another RCT of VC/VS DBS in eight TRD patients was discontinued after an interim futility analysis of active vs. sham stimulation showed no difference in effects between the two groups after 16 weeks. These results were never published but were discussed by Rezai et al. [70].

### 3.4. The Ventral Part of the Anterior Limb of the Internal Capsule

The anterior limb of the internal capsule (ALIC) is another brain region that was initially studied for DBS in OCD. One study aimed at stimulating the NAcc discovered that most treated OCD patients (9 out of 16) actually received DBS in the ventral part of the ALIC (vALIC), which improved obsessive compulsive scale scores, showed anti-depressive effects, and led to the clinical implementation of vALIC-DBS in TRD [29]. DBS of the vALIC has also been associated with a decreased metabolism in the OFC, subgenual anterior cingulate cortex, and right dlPFC [71,72,73] (Table 2).

The first RCT of DBS of the vALIC for TRD was conducted by Bergfeld et al. (2016), investigating 25 TRD patients during a 52 week open-label trial, which resulted in a significant decrease in HDRS scores in the whole group during the optimization phase, although overall HDRS scores were still in the depression range (22.2 at baseline vs. 15.9 after optimization phase). Ten of the 25 patients could be classified as responders, with a more than 50% decrease on the HDRS. After the optimization phase, a RCT with a cross-over design including nine responders and seven non-responders ensued and showed a significantly lower score in the active DBS phase compared with the sham DBS phase (mean HDRS score of 13.6 (95% CI; 9.8–17.4 vs. 23.1 (95% CI; 20.6–25.6)) (HDRS < 0.001). However, the scores on the HDRS in the active treatment group were still within the mild to moderate depression range [74]. Both crossover phases lasted approximately 21 and 18 days, respectively. 

### 3.5. Lateral Habenula

The activity of the lateral habenula (LHb) is negatively associated with reward, meaning its neurons increase their firing rate in a non-reward situation or in the omission of a reward. LHb hyperactivity could therefore explain the lower reward-seeking behavior in TRD [75] (Table 2). Speculation that DBS of the LHb could lead to the inhibition of hyperactivity prompted the first case study of LHb-DBS in TRD, which notably led to full remission of the patients’ depressive symptoms [44]. A clinical non-randomized study in six patients suffering from TRD is currently being held, investigating the safety, tolerability, and benefit of LHb DBS in TRD. Patients that respond at 12 months of stimulation will enter a randomized, staggered withdrawal phase. During this phase, a double-blind discontinuation will be attempted at month 12 or 13, decreasing the stimulation by 50% and then completely discontinuing it during the following two weeks. Evaluation will take place at 15 months, where, in the meantime, escape criteria are included, and if met, will stop the blinded phase in continuing with an open treatment [76].

### 3.6. Thalamic Peduncles

The inferior thalamic peduncle (ITP) is a bundle of fibers connecting the OFC to the thalamus. The OFC is thought to play a role in the non-reward tractor theory of depression, where the orbitofrontal non-reward system is more easily triggered in depression, causing negative emotional states [77] (Table 2). Stimulating the ITP could disrupt this enhanced triggering and lead to less depressive symptoms. ITP stimulation for OCD has already shown improvements of the score on the Yale-Brown Obsessive-Compulsive scale in five OCD patients [45]. A case study in one TRD patient reported that DBS of the ITP decreased depressive symptoms [78]. However, within this study, two brain regions were investigated, the second being the BNST. 

### 3.7. Bed Nucleus of the Stria Terminalis

The BNST is involved in a range of behaviors, such as stress response, social behavior, and extended duration of fear states. This nucleus assesses sensory information from the environment, coupled together with the subjects current mood and arousal, integrating a proper response to environmental and social setting changes [22] (Table 2). Raymaekers et al. (2017) indicated that both BNST and ITP stimulation could alleviate depressive symptoms; however, due to a small sample size, no statistical analyses were conducted [78]. 

### 3.8. Medial Forebrain Bundle

The medial forebrain bundle (MFB) is a fiber tract connected to various parts of the limbic system thought to play a role in reward-seeking systems [21] (Table 2). In one trial, DBS of the superolateral branch of the MFB resulted in more than a 50% decrease in depressive symptoms in six out of seven TRD patients within seven days [47]. An additional interim analysis of MFB-DBS in TRD confirmed these findings, showing more than a 50% decrease in depressive symptoms in three out of four patients within seven days of stimulation. At 26 weeks follow-up, two patients showed more than an 80% decrease in depression rating scales [49] (Table 1).

Taken together, the results of the aforementioned studies of DBS for TRD imply that stimulation at a number of different brain areas can alleviate depressive symptoms, which is in line with the view that MDD is a circuitopathy involving various brain regions and networks mainly within the limbic CSTC mood circuits [12,79]. However, how DBS of those targets improves the depressive symptoms is not completely clear. Moreover, stimulation parameters vary between studies due to a need to adjust and balance therapeutic effects to side effects.

MDD is a circuitopathy that involves a wide range of brain structures and exhibits diverse clinical manifestations. Therefore, a one-size-fits-all approach to the DBS targeting may not be beneficial in all patients, whereas a patient-centric selection based on individually disrupted neurocircuits could improve therapeutic outcomes. In evaluating the effects of DBS, one needs to focus on overall improvement on depression rating scales as well as individual scores and symptom-specific improvements. This will enhance the understanding of the effects of DBS and eventually contribute to the development of more personalized treatment approaches. Seemingly, this also applies in other psychiatric disorders such as OCD, where personalized approaches with content-specific DBS targets have already proven to be beneficial [80]. 

## 4. Towards a More Personalized DBS Treatment Approach for Treatment-Resistant Depression

Since open-label trials and RCT data on DBS in TRD show inconsistent results, this gives rise to discussion about the chosen study designs, the correct interpretation of results, and the best target(s) for neuromodulation. Depression entails different clinical subtypes and looking at homogenous subgroups of depressed patients may lead to a personalized DBS approach. This would be superior to looking at primary outcomes across all participants. Importantly, a prerequisite to this approach is the ability to determine pathoanatomical substrates of specific subtypes. How to implement such a more personalized approach to DBS treatment for TRD is discussed below.

### 4.1. Clinical and Neurophysiological Subtypes of Depression

Most response rates in depression treatments to date have been measured with changes in average levels among all patients treated. However, depressive symptomatology varies highly among individuals, making the standardization of positive outcomes challenging. Mood, sleep rhythm, concentration, psychomotor, and cognitive domains can all be disturbed in depression, while treating one selected brain structure within the mood circuit may not have an effect on all aforementioned symptoms nor have an effect on the main symptomatology of all depressed patients.

Subdividing TRD into different subtypes, involving distinct clinical symptoms as well as distinct patterns of dysfunctional connectivity in limbic and frontal striatal networks, may reveal different subtype-related outcomes for each investigated brain region, and if so, patient selection for a given brain target could enhance treatment effectiveness [81]. Analysis of resting-state connectivity biomarkers previously revealed four connectivity-based biotypes of depression characterized by either anxiety, increased anhedonia, psychomotor retardation, and/or increased anergia and fatigue. Moreover, patients could not be differentiated into a particular subtype based on clinical features alone and clustering them based on functional connectivity was needed [82]. Therefore, imaging procedures as well as featured symptoms should be taken into account when treating TRD with DBS. It is conceivable that subdividing TRD patients according to connectivity-based biotypes will shed new light on the interpretation of previous DBS study results, and that the integration of functional connectivity in future DBS studies will reveal clinically relevant subgroups that might respond to DBS of a specific target within the mood circuit. Altogether, it can be suggested that better assessment of therapeutic outcomes at symptom level might be accomplished when TRD patients with dominant anergia/fatigue symptoms (biotype 2) are stimulated within the CG 25; and patients characterized by more anxiety (biotype 4) are stimulated within the thalamic region, as suggested by Drysdale and coworkers [82]. Likewise, SCG stimulation could alleviate sleep disturbances and NAcc stimulation could improve anhedonia (Table 2).

### 4.2. Individual Tractography

Another way in which DBS efficiency can be improved is to ameliorate the implantation of electrodes with the usage of individualized, patient-specific, deterministic tractography targeting. Riva-Posse et al. (2018) used individualized patient-specific tractography targeting for SCC-DBS surgeries in TRD patients, aiming at the convergence of the four white matter bundles: the forceps minor, uncinate fasciculus, cingulum, and fronto-striatal fibers. This resulted in a response rate of 81.8% and a remission rate of 54% after a one year trial period, which proved greater than the previous open-label trials [83]. In a recent study, diffusion tensor imaging (DTI) tractography was used to target SCC-DBS more optimally, and the authors examined the impact of tract activation on clinical response at 6 and 12 months. Stimulation of vmPFC pathways by SCC-DBS was associated with a positive response and stimulation of the cingulum was associated with a 6 month, but not a 12 months DBS response. Monopolar stimulation of 130 Hz was applied with either pulse width (90–450 µs) or amplitude (4–8 V) progressively increased every month, based on response status. Patients were changed to bipolar settings if monopolar stimulation caused adverse effects. It was speculated that targeting more ventral, rather than the dorsal mPFC projections, might improve the response [84].

### 4.3. Combining Deep Brain Stimulation with Cognitive-Behavioral Therapy

It is plausible that better therapeutic outcomes could be achieved if DBS is applied in combination with concurrent treatments, such as pharmacotherapy with antidepressants or cognitive-behavioral therapy (CBT) in TRD. Studies focusing on the added effect of concurrent treatments to DBS have not been conducted in patients with TRD. The results from studies in OCD patients treated with DBS show that adding CBT to DBS has added beneficial effects [85]. Studies targeted at revealing the added effects of concomitant treatments after DBS for TRD would also provide information that may facilitate establishing a treatment algorithm to determine the place of these treatments in DBS patients. 

### 4.4. Biomarkers

Biomarkers are quantifiable characteristics of biological processes, which could prove helpful in improving diagnostic objectivity of MDD and TRD as well as help in personalizing its treatment. For MDD, no specific biomarkers have yet been found, though several markers have been shown to be potential candidates, such as brain-derived neurotrophic factor (BDNF), interleukins (IL) 1 and 6, tumor necrosis factor (TNF), malondialdehyde (MDA), hypothalamic-pituitary-adrenal (HPA) activity, and cortisol responses [86,87]. Every biomarker as a standalone shows a low sensitivity and specificity, partly explained by the heterogeneity of MDD. To overcome this shortcoming, either examining a biological panel of several markers [88] or phenotyping MDD and TRD into distinct subtypes could be considered. However, a recent meta-analysis showed that only cortisol has a predictive effect on onset/relapse and recurrence of MDD making the integration of biomarkers for personalizing TRD treatment a futuristic milestone yet to be discovered [89]. 

### 4.5. Insights into Symptomatic Improvement after Deep Brain Stimulation

For TRD, different regions in the mood circuit can be stimulated with DBS (Table 2), although it is still unclear which depressive-symptoms respond to the stimulation of a specific target. More research into the mood circuit is needed to untangle which emotions arise from specific brain regions. This may vary from basic animal research, disentangling neuronal function per brain region, and ultra-high field MR studies in humans, all of which could shed light on the dysfunctional brain circuits in TRD. In contrast to the motor system that is studied thoroughly [90,91], emotional circuitry is far less understood. One reason for this is that animal research into mood circuitry remains complicated as there is considerable heterogeneity between species [92]. Modeling depression in animals is complex as there are several depressive-like behavior models, such as the chronic unpredictable stress paradigm (CUS), which give insight into depression pathology [93]. DBS is investigated within these models to unravel behavioral and cellular changes following DBS [94]. 

Alongside the standard clinical rating scales, the use of momentary assessment techniques, such as the experience sampling method (ESM), could enhance the documentation of the momentary mood states [95]. The ESM includes short repeated assessments of experiences and behaviors, as well as moment-to-moment changes in mental states in the context of daily life. Research has shown that depressed patients can improve their depressive symptomology while using weekly ESM for six weeks, and add-on ESM derived feedback resulted in a significant decrease in HDRS scores compared to controls (*p* < 0.01; −5.5 point reduction in HDRS at 6 months) [96]. In add-on-derived feedback, a psychologist or psychiatrist gives feedback on the association between the participants momentary affective states and specific daily life contexts [97]. ESM-derived feedback could further improve treatment by showing within-subject changes in a heterogeneous TRD population and contribute to clinical decision-making [97]. In the case of DBS, the use of ESM may reveal specific response patterns depending on the brain region that is stimulated, which can provide valuable information about emotional circuitry. This can be done using well-evaluated day-to-day scores, including questionnaires that go into detail on current mood and adaptive functioning.

## 5. Conclusions

More personalized treatment approaches hold the potential to increase the overall efficacy of DBS for TRD. Precise evaluations of symptoms, biomarkers, and resting-state connectivity patterns are essential when distinguishing clinical subtypes of TRD. Moreover, subtyping may provide more insight into the working mechanisms of DBS and help in selecting optimal targets in patients. Monitoring of biomarkers at multiple time points during treatment along with evaluation of ESM data, in parallel with clinical assessments of mood using standardized depression-rating scales, will lead to a better understanding of symptom changes when stimulating specific brain regions. Such considerations could further lead to optimal adjustments of stimulation parameters as long-term effects of DBS on mood occur. 

## Figures and Tables

**Table 1:** DBS in treatment-resistant depression (TRD); published open-label and randomized clinical trials.

Region (DBS)	Study	Open-Labeled, RCT or Case-Report	N	Follow-Up	Age (Mean)	Length of Current Depressive Episode, Years (Mean)	Response Rate (%) in HDRS or MADRS Scores	Remission Rate (%)	Serious Adverse Events (N)
SCG	Mayberg et al., 2005	Open-label	6	6 months	46	5.58	33.3 (1 month), # 83 (2 months), # 66.6 (6 months), #	0 (1 month) 33.3 (3 months) 33.3 (6 months)	Suicidal ideation: 2 Syncope: 1 Lead problem: 1
	Lozano et al., 2008	Open-label	20	12 months	47.4	6.9	60 (6 months), # 55 (12 months), #	35 (6 months) 35 (12 months)	Seizure: 1 Lead problem: 3
	Kennedy et al., 2011	Open-label	20	1, 2 and 3 years, last follow-up (3–6 years)	47.4	6.9	62.5 (1 year), # 46.2 (2 years), # 75 (3 years), # 64.3 (last follow-up), #	18.8 (1 year) 15.4 (2 years) 50 (3 years)	Worsening depression:3 Suicidal ideation:3
	Puigdemont et al., 2012	Open-label	8	12 months	47.4	6.3	87.5 (1 week), # 37.5 (1 month), # 87.5 (6 months), # 62.5 (12 months), #	50 (1 week) 37.5 (6 months) 50 (12 months)	Suicide attempt: 1
	Lozano et al., 2012	Open-label	21	12 months	47.3	5.0	57 (1 month), # 48 (6 months), # 29 (12 months), #	-	Suicide: 1 Suicide attempt: 1
	Holtzheimer et al., 2012	Open-label	17	24 months	42	5.34	41 (6 months), # 36 (12 months), # 92 (24 months), #	18 (6 months) 36 (12 months) 58 (24 months)	Suicidal ideation: 1 Suicide attempt: 2
	Merkl et al., 2013	Open-label	6	24 h Last follow up (24–36 weeks)	50.66	2.13	33.33 (36 weeks), #	33.33 (36 weeks)	Headaches: 6 Tenseness in neck region: 1
	Holtzheimer et al., 2017	*RCT*	60 (52)	6 months (24 months)	50.53	12.62	22 (6 months), ‡ 54 (12 months), ‡ 48 (24 months), ‡	10 (6 months) 17 (12 months) 25 (24 months)	Suicide attempt: 2 Suicidal ideation: 2 Seizure: 2
	Eitan et al., 2018	*RCT HF* vs. *LF DBS*	9	13 months	46	-	44.44 (13 months), ‡	-	-
	Merkl et al., 2018	RCT	8	28 months (*n* = 6) 4 years (*n* = 2)	48.25	2	37.5 (6 months), # 43.0 (12 months), # 23.0 (28 months), #	12.5 (6 months) 14.2 (12 months) 33.0 (24 months) 33.3 (28 months)	Manic episode: 1
	Crowell et al., 2019	Open-label	28	4 (*n* = 14) 8 (*n* = 11) years	44.9 (45.9)	45.1 (46.6)	18 #	21	Suicide attempt: 6 Suicidal ideation: 8 Anxiety: 6 Worsening depression: 2
PGR	Accolla et al., 2016	Open-label	5 (1)	6 months (24 months)	45.20	-	-	-	-
NAcc	Schlaepfer et al., 2008	Open-label	3	6–24 weeks	46.7	7.2	-	-	None
	Bewernick et al., 2010	Open-label	10	10 months	48.6	10.8	50 (1 month), # 50 (6 months), # 50 (12 months), #	30 (1 month)	Suicide: 1 Suicide attempt: 1
	Bewernick et al., 2012	Open-label	11	12 months 24 months Last follow up (max 4 years)	48.36	9.26	45 (12 months), #	9.1 (24 months)	Pain: 4 Seizure: 1 Agitation: 3 Suicide:1 Suicide attempt: 1
VC/VS	Malone et al., 2009	Open-label	15	6 months (*n* = 15) 12 months (*n* = 11)	46.3	21	20 (1 month), # 40 (6 months), # 53.3 (last follow-up) #	20 (6 months) 40 (last follow-up)	Suicidal ideation: 2 Syncope: 1 Lead problem: 1
	Dougherty et al., 2015	RCT	30	24 months	47.7	11.4	20 (16 weeks), ¥ 20 (12 months), ¥ 23.3 (24 months), ¥	13 (12 months) 20 (24 months)	Suicide: 1 (stimulation off) Suicide attempt: 4 Suicidal ideation: 5 Lead revision: 3
vALIC	Van der Wal et al., 2020 (follow-up of the RCT Bergfeld et al. 2016)	Open-label	25	2 years	52.5	7.42	32.0 (2 years, ITT) #	20.0 (2 years, ITT)	Pain: 1 Agitation: 3 Suicidal ideation: 6 Fatigue: 4
	Bergfeld et al., 2016	RCT	25	52 weeks	53.2	6.98	40 (after optimization of DBS settings (T_2_)) #	20 (T_2_)	Suicide attempt: 4 Suicidal ideation: 3 Automutilation: 1
LHb	Sartorius et al., 2009	Case-report	1	60 weeks	64.0	9.0	-	-	-
MFB	Schlaepfer et al., 2013	Open-label	7	12–33 weeks	42.6	7.6	86, ¥	57	Cranial bleeding: 1
	Fenoy et al., 2016 (interim analysis)	Open-label	4	52 weeks	-	-	75 (7 days) ¥66 (26 weeks, OC) ¥	-	-

“-”: has not been mentioned in this article, RCT; response criteria; #; 50% or greater reduction in Hamilton Depression Rating Scale (HDRS) _(17 or 28)_ scores, ¥; 50% or greater reduction in MADRS scores, ‡; 40% or greater reduction in MADRS scores, RCT; randomized controlled trial, ITT; intention to treat, OC; observed case.

**Table 2:** Targets for DBS in treatment resistant depression (TRD), functions, pathophysiology and the effect of DBS.

Brain Region	Function	Pathological Activity in MDD	HF-DBS Effect
SCG	Contains three white matter bundles; forceps minor + uncinate fasciculus connecting to the medial frontal cortex, cingulum connecting to the rostral and dorsal ACC and fronto-striatal fibers connecting to the NAcc, CN, Pt and anterior Th Connects higher ‘top-down’ cortical regions with subcortical modulatory regions Involvement in brain DMN [30]	Increased activity [31] Reduced volume in familial depression [32] Projections to: (1) NAcc may play a role in lack of interest, disruption in reward and underlie anhedonia (2) Hth and brainstem may play a role in circadian and sleep disturbances, problems with appetite and an abnormal stress responds and cortisol metabolism [31].	Disruption of pathological activity Modulation of multiple regions connected to the SCG [31]
NAcc	Receives projections from VTA, AG, OFC, mPFC, dCN, GP and Hip and projects to Cg25, mPFC, VP, Th, AG and Hth. Transmits information from emotion centers to motor control regions, causing motivational behavior to obtain rewards [33] Processes reward and pleasure information	In severe anhedonia; smaller size and less activation to reward [34]	Acute: Increase in exploratory motivation Chronic: reduction in anhedonia PET Imaging: ↑ activity in VS, bilateral dlPFC and dmPFC, cingulate cortex and bilateral AG. ↓ activity in vmPFC and vlPFC, dCN and Th [33]
VC/VS	Contains fibers connecting the dPFC, dACC, OFC and vmPFC with THAL, AG, Hth and brainstem (SN, VTA, RN and PTN) [35]	Increased activity [36] Activation of the connection from left vs. to left caudate has been associated with anhedonia Increased connectivity of vs. to DMN is positively correlated to higher depression scores in the CES-D score [37]	-
vALIC	Contains two fiber bundles: the anterior thalamic radiation and the supero-lateral branch of the MFB connecting the PFC to different subcortical structures such as the Th, NAcc, VTA and VS. Decreased integrity of the right vALIC in depressed patients [38]	-	Decreased metabolism in OFC, subgenual ACC and right DLPFC in patients with OCD [39]
LHb	Activity corresponds negatively to anticipation and reception of a reward [40]	Increased activity [41] Possible down regulation of serotonergic, noradrenergic and dopaminergic systems [42], volume reduction [43]	Localized metabolic increase in one patient with FDG-PET, presumably due to functional inhibition [44]
ITP	Interconnects the intralaminar nucleus and TRN with the OFC [30,45]	Hyperactivation in both TRN and OFC [46]	Cortical desynchronization Disruption of adrenergic and serotonergic malfunction [46]
MFB	Interconnects the Nacc, VTA, vmHth, lHth and AG ventromedial and lateral nuclei of the Hth and AG with convergence onto the PFC [47,48] Plays a crucial role in the reward pathway;	Dysfunctional reward system. Responders showed a strong connectivity between the active electrode contact and the mPFC pre-operatively using individual DTI [49]	Insignificant changes in metabolism in 3 patients with PET measurements pre-operatively, 6 and 12 months post-operatively [49]
BNST	Mayor output pathway of the AG Regulates stress response Integrates information from multiple brain areas to perform ‘valence surveillance’ [22,30]	Oscillatory activity with high a-power [50]	-

“-“: not investigated; (d) ACC; (dorsal) anterior cingulate cortex, AG; amygdala, CES-D score; center for epidemiologic studies depression score, (d) CN; (dorsal) caudate nucleus, DMN; default mode network, DTI; deterministic diffusion tensor imaging, GP; globus pallidus, Hip; hippocampus, Hth; hypothalamus, MFB; medial forebrain bundle, OFC; Orbitofrontal cortex, (m/dl/dm/vm/vl) PFC (medial/dorsolateral/dorsomedial/ventromedial/ventrolateral) Prefrontal cortex, PGR; posterior gyrus rectus, PTN; pedunculopontine tegmental nucleus, Pt: putamen, RN; raphe nuclei, SN; substantia nigra, Th; thalamus, TRN; thalamic reticular nucleus, VP; ventral pallidum, VS; ventral striatum, VTA; ventral tegmental area.
